# Supplementary material for: The Distinction between Dematiaceous Molds and Non-Dematiaceous Fungi in Clinical and Spiked Samples Treated with Hydrogen Peroxide Using Direct Fluorescence Microscopy
Source: J Fungi (Basel). 2023 Feb 9;9(2):227. doi: 10.3390/jof9020227 (PMC9967646; doi:10.3390/jof9020227)
Supplement: Supplementary file 1 [file jof-09-00227-s001.zip › Table S1_Final.pdf]

Table S1. Preparation of smears, microscopic evaluation and image recording in the study

**1**	**Control smears**		**Images recorded at 2 magnifications (x200, x400)**
**1.1**	No stain or other additives	1.1.1	Bright field
		1.1.2	1st fluorescence filter
		1.1.3	2nd fluorescence filter
**1.2**	Calcofluor white 0.1% stain	1.2.1	Fluorescence microscope
**2**	**Test smears**		**Images recorded at 2 magnifications (x200, x400)**
**2.1**	10% Hydrogen peroxide added	2.1.1	Bright field
		2.1.2	1st fluorescence filter
		2.1.3	2nd fluorescence filter
**2.2**	30% Hydrogen peroxide added	2.2.1	Bright field
		2.2.2	1st fluorescence filter
		2.2.3	2nd fluorescence filter
